# Predicting lung cancer survival with attention-based CT slices combination

**DOI:** 10.1007/s13755-025-00404-z

**Published:** 2026-01-04

**Authors:** Domenico Paolo, Carlo Greco, Edy Ippolito, Michele Fiore, Sara Ramella, Paolo Soda, Matteo Tortora, Alessandro Bria, Rosa Sicilia

**Affiliations:** 1 Unit of Artificial Intelligence and Computer Systems, Department of Engineering, University Campus Bio-Medico of Rome, Rome, Italy; 2 Department of Naval, Electrical, Electronics and Telecommunications Engineering, University of Genoa, Genoa, Italy; 3 Department of Diagnostics and Intervention, Radiation Physics, Biomedical Engineering, Umeå University, Umeå, Sweden; 4 Department of Electrical and Information Engineering, University of Cassino and Southern Latium, Cassino, Italy; 5 Department of Medicine and Surgery, Research Unit of Radiation Oncology, Università Campus Bio-Medico di Roma, Rome, Italy; 6 Operative Research Unit of Radiation Oncology, Università Campus Bio-Medico di Roma, Rome, Italy

**Keywords:** Deep learning, Non-small cell lung cancer, Overall survival, Risk analysis, Soft-attention

## Abstract

Accurate prognosis of Non-Small Cell Lung Cancer (NSCLC) is crucial for enhancing patient care and treatment outcomes. Despite the advancements in deep learning, the task of overall survival prediction in NSCLC has not fully leveraged these techniques, yet. This study introduces a novel methodology for predicting 2-year overall survival (OS) in NSCLC patients using CT scans. Our approach integrates CT scan representations produced by EfficientNetB0 with a soft attention mechanism to identify the most relevant slices for survival risk prediction, which are then analyzed by a risk-assessment network. To validate our method and ensure reproducibility, we employed the public LUNG1 dataset and a smaller private dataset. Our approach was compared to benchmark 3D networks and two variants of our methodology: on the LUNG1 it outperformed the competitors achieving a mean $C^{td}$-index of 0.584 over tenfold cross-validation. On the LUNG1 we also demonstrated the adaptability of our method with 5 other 2D backbones replacing the EfficientNetB0, confirming that our mechanism of combining 2D slice representations to construct a 3D volume representation is more effective for OS prediction compared to a traditional 3D approach. Finally, we used transfer learning on the private dataset, showing that it can significantly enhance performance in limited data scenarios, increasing the $C^{td}$-index by 0.076 compared to model without transfer learning.

## Introduction

Lung cancer remains one of the leading causes of cancer-related mortality worldwide, making early diagnosis and accurate prognosis critical for improving patient outcomes [1]. Computed tomography (CT) is the standard imaging modality for lung cancer detection and monitoring, providing high-resolution, volumetric information about tumor morphology and surrounding tissues. CT imaging is widely available in clinical practice and offers detailed structural insights that are essential for treatment planning and disease progression assessment.

Integrating artificial intelligence (AI), and in particular deep learning methods, into CT imaging analysis has the potential to further enhance clinical decision-making. Convolutional neural networks (CNNs) have become the backbone of state-of-the-art approaches for medical image analysis, enabling automated detection, segmentation, and characterization of lung lesions [2–4]. The increasing availability of annotated CT datasets has facilitated the development of models capable of assisting radiologists and clinicians in diagnosis and prognosis.

Despite these advancements, predicting overall survival (OS) from CT images remains challenging. OS prediction involves estimating the likelihood that a patient will survive over a defined timeframe and requires capturing subtle imaging patterns correlated with patient outcomes. Developing models for OS prediction is difficult due to the high computational demands and extensive data required to process complex medical images like CT scans [5]. These models often exhibit limited generalization capability due to the inherent variability in medical images, particularly when applied across different institutions and equipment, resulting in reduced robustness and accuracy. Moreover, reliance on large annotated datasets presents practical and ethical challenges, while variations in image quality and annotation consistency can further complicate model training and introduce noise.

To fill this gap, in this work, we propose a novel methodology that leverages CNN representations of CT scans, integrated through a soft attention mechanism to highlight the most relevant slices in the volume for the 2-year OS prediction task in Non-Small Cell Lung Cancer (NSCLC) patients. NSCLC is the most prevalent form of lung cancer, accounting for an estimated 135,000 deaths annually [6]. Accurate prognosis is crucial for effective treatment planning and improved patient care. We focus on a 2-year OS because this timeframe serves as a critical window for prognostication in NSCLC patients, allowing physicians to evaluate the effectiveness of initial treatments and to tailor therapeutic strategies based on the individual patient’s response [7–11].

Our main contributions are summarized as follows:

- We propose a completely automated process for extracting a rich 3D representation from CT data by utilizing a 2D CNN backbone combined with a soft attention mechanism;
- We validate our approach on a real-world clinical problem, conducting ablation studies to assess the effectiveness of the soft attention mechanism in assigning varying importance to each slice of the 3D volume and employing the time-dependent concordance index ($C^{td}$-index) as a performance metric to capture the evolution of risk over time;
- The results show that the proposed approach outperform conventional 3D networks in producing robust 3D representations for OS prediction, demonstrate its adaptability across diverse 2D backbone architectures, and underscore the critical importance of a dual-stage transfer learning strategy to effectively address challenges posed by data-limited scenarios.

The rest of this manuscript is organized as follows: Section "Related works" reviews related works. Section "Materials" introduces the datasets, providing an overview of the adopted pre-processing steps. Section "Methods" describes the details of our approach. Section "Experimental setup" explains the experimental setup, while Section "Results and Discussion" discusses the results. Finally, Section "Conclusions" offers concluding remarks.

## Related works

With the advent of high-quality CT scans, it has become feasible to integrate quantitative image features into OS prediction models. OS prediction involves estimating how long patients are expected to live after being diagnosed with a disease. This task can be divided into two main categories: OS classification and OS regression. In OS classification, the goal is to predict whether a patient will survive beyond a specific time threshold, framing the problem as a classification task with a categorical outcome. On the other hand, OS regression focuses on estimating the precise time until the event of interest (death) occurs, while accounting for censored data, i.e. data where the event of interest has not been observed for certain subjects by the end of the study period. In this case, the task is to predict how many months or years a patient is expected to survive after diagnosis, treating the problem as a regression task where the target is a continuous variable (time to event). Traditionally, such studies have primarily relied on radiomics features, which consist of a predetermined set of mathematically defined characteristics [12–14]. However, AI-based techniques, particularly deep learning approaches, have emerged as valuable tools for the automatic learning of potentially relevant patterns from medical images [15]. Recent studies [7–11] have explored the use of deep learning for OS classification in NSCLC patients using the public NSCLC-Radiomics (LUNG1) dataset from the MAASTRO clinic [16], which, to the best of our knowledge, represents the most comprehensive and extensive resource for OS prediction in NSCLC cases.

In Braghetto et al. [7], radiomics and deep-learning approaches were compared on the LUNG1 dataset for the classification of 2-year OS. For radiomics, the study considered the best combination between two feature selectors (ANOVA, Cluster reducer) and six classifiers (SVM, BAG, XGB, Neural Network, KNN, RF). The optimal combination achieved an average AUC of 0.67 across five random test splits. The deep learning approach, consisting of a 2D convolutional neural network (CNN), achieved a slightly lower average AUC of 0.64 across the same splits. In Zheng et al. [8], a hybrid model that integrated both image and clinical features was implemented using a 3D CNN for the classification of 2-year OS in stage I-IIIA non-small cell lung cancer patients. Image features were learned from cubic patches containing lung tumors extracted from pre-treatment CT scans. Relevant clinical variables were identified through analyses, revealing that age and clinical stage are the most significant prognostic factors for 2-year OS. Using these two clinical variables in combination with image features from pretreatment CT scans, the hybrid model, after training on the University Medical Center Groningen (UMCG) dataset, achieved a median AUC of 0.64 on the LUNG1 test set, which contained 228 patients ($n = 228$) with stage I-IIIA lung tumors treated with radiation or concurrent chemoradiation. In [9], a foundation model for cancer 2-year OS classification was developed by training a convolutional encoder through self-supervised learning using a comprehensive dataset of 11,467 radiographic lesions. The foundation model was then fine-tuned using data from the HarvardRT cohort ($n = 291$) to classify 2-year overall survival after treatment. Subsequently, the model was evaluated on the LUNG1 cohort, achieving an AUC of 0.638.

Deep learning techniques have also been applied on LUNG1 for OS regression. In [10] Torres et al. developed a fully automated imaging-based prognostication technique (IPRO) using a 3D CNN to predict 1-year, 2-year, and 5-year mortality from pretreatment CTs of patients with stage I-IV lung cancer by sing six publicly available data sets (1,689 patients, of whom 1,110 were diagnosed with non–small-cell lung) [16–21]. IPRO showed a Concordance-index (C-index) of 0.72 for 1-year, 0.70 for 2-year, and 0.68 for 5-year mortality, performing a fivefold cross-validation. In [11], Haamburger et al. showed that by simplifying survival analysis to median survival classification, convolutional neural networks can be trained with small batch sizes and learn features that concatenated with radiomics features predict survival equally well as end-to-end hazard prediction networks. They obtained a C-index of 0.623 over 100 random splits, where for each split, 60%, 15% and 25% of the data was used for training, validation and testing, respectively. Direct hazard predictions from a neural network with radiomics features (multi-modal) and without were less precise with C-index of 0.613 and 0.585 respectively.

While these studies have demonstrated promising results in both classification and regression tasks, there are several notable limitations. A major limitation is represented by the high dimensionality of CT data and model complexity. The use of 3D CNNs in survival prediction models, as in studies like Zheng et al. [8] and Torres et al. [10], involves processing vast amounts of image data. Although 3D CNNs can capture volumetric details, the high dimensionality leads to increased computational costs and the risk of overfitting, particularly when datasets are limited in size. In this work, we propose a method that integrates a 2D convolutional neural network (CNN) with a soft attention mechanism to create a comprehensive representation of the 3D volume for the 2-year OS regression task. This approach aims to leverage the benefits of automatic feature extraction from images while focusing on relevant slices within the 3D images, which is achieved through the attention mechanism. Using 2D CNNs with soft attention reduces the complexity of the model compared to 3D CNNs, which can lead to easier training and better generalization, especially when data is limited. Another limitation consists in the underutilization of temporal dynamics in survival prediction since these state-of-the-art papers primarily focus on fixed time points (e.g., 2-year survival), not accounting for the dynamic nature of survival, where the risk of mortality changes over time. In this work, we address this issue by processing the enhanced representation, generated by a CNN combined with a soft attention mechanism, through a risk-assessment network known as DeepHit [22]. This network is specifically designed to manage censored data, a common challenge in medical research. Censored data refers to patients whose follow-up ended before the completion of the 2-year observation period. In survival analysis, these patients provide partial information: we know only that they survived until their last follow-up.

DeepHit employs a specialized loss function that not only accounts for censoring but also optimizes survival predictions over multiple time points, rather than a single static measure, making it particularly well-suited for predicting OS. By utilizing this method, we are able to estimate survival risk at several points across the 2-year time horizon, providing a dynamic, rather than static, survival prediction. To assess the effectiveness of the proposed approach, we employ the time-dependent C-index ($C^{td}$-index), which is particularly suitable for OS regression as it accounts for the time to the event. This index offers a more comprehensive evaluation of model performance over the entire time horizon, rather than limiting the assessment to a single endpoint. Consequently, it provides deeper insights into the model’s ability to predict survival risks at different intervals, ultimately leading to more robust and accurate survival predictions.

## Materials

We used two datasets for our experiments: the publicly available LUNG1 dataset [16] and a proprietary dataset, hereafter referred to as CLARO. The LUNG1 dataset consists of 422 patients with stages I-III NSCLC. We excluded seven patients from the 422 due to missing CT slices in the complete volume (i.e. LUNG1-014, LUNG1-021, LUNG1-085, LUNG1-095, LUNG1-128, LUNG1-194, LUNG1-246). The CLARO dataset consists of 119 patients with stage III NSCLC, who received concurrent radiochemotherapy at the radiotherapy department of Campus Bio-Medico University, and has already been utilized in our previous studies [23, 24].

CT scans in LUNG1 were acquired on different Siemens Sensation models (16, 40, 10, and Open) and reconstructed at 3 mm slice thickness [16], whereas the CLARO scans were obtained on a Siemens Somatom Emotion scanner with fixed acquisition parameters (140 kV, 80 mAs, 3 mm slice thickness) and standardized reconstruction kernels (B70 for lung, B31 for mediastinum) [25]. These differences in scanner hardware and reconstruction protocols may affect image characteristics such as sharpness, noise, and contrast, thereby contributing to domain shift.

The 2-year OS distributions of the two datasets are depicted in Table 1. For both datasets, we employed only the pretreatment CT scans. The preprocessing steps applied to these scans are outlined in the following subsection.

**Table 1:** 2-Year OS distributions for LUNG1 and CLARO datasets

Dataset	Outcome	A-priori probability
LUNG1	0 (Survivor)	0.40
	1 (Non-Survivor)	0.60
CLARO	0 (Survivor)	0.57
	1 (Non-Survivor)	0.43

**Fig. 1:**
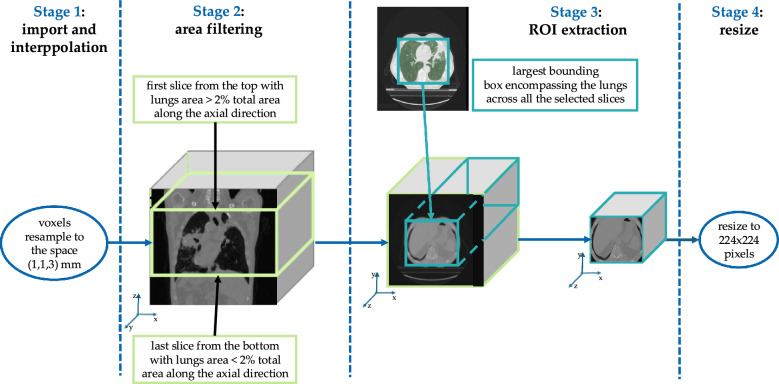
Preprocessing Pipeline

### Preprocessing

The preprocessing pipeline of a single CT scan is depicted in Fig. 1. In the initial step, we extracted a 3D array with voxel intensity values represented as Hounsfield Units from Digital Imaging and Communications in Medicine (DICOM) data and resampled the image voxels to a resolution of (1, 1, 3) mm to standardize the spatial dimensions of the CT scan. This resolution strikes a balance between spatial detail and computational efficiency. A voxel size of 1 mm in the x and y directions ensures high spatial resolution in the axial plane, which is essential for accurately visualizing fine details of lung tumors and lesions. Meanwhile, the 3 mm resolution in the z direction reduces the number of slices needed to cover the entire volume, thereby lowering computational demands without significantly sacrificing diagnostic quality.

Next, we applied an area filtering criterion to isolate a specific volume of the CT scan. This step utilized a U-net model, trained specifically for lung segmentation [26], to segment each individual slice and extract the right and left lungs. Slices containing less than 2% lung area relative to the total slice area were excluded to remove apical or basal sections with minimal lung tissue. This threshold was empirically chosen to balance prognostically relevant regions and computational efficiency, as processing entire CT volumes with full peripheral slices would significantly increase GPU memory usage.

In the third step, we focused on extracting the region of interest (ROI). Once all slices were segmented, we extracted the ROI by identifying the largest bounding box that encompassed the lungs across all the selected slices and applied it to the entire volume.

Finally, we resized each slice to $224\times 224$ pixels to standardize the input size for subsequent processing stages, thus enabling the use of pre-trained models on ImageNet.

**Fig. 2:**
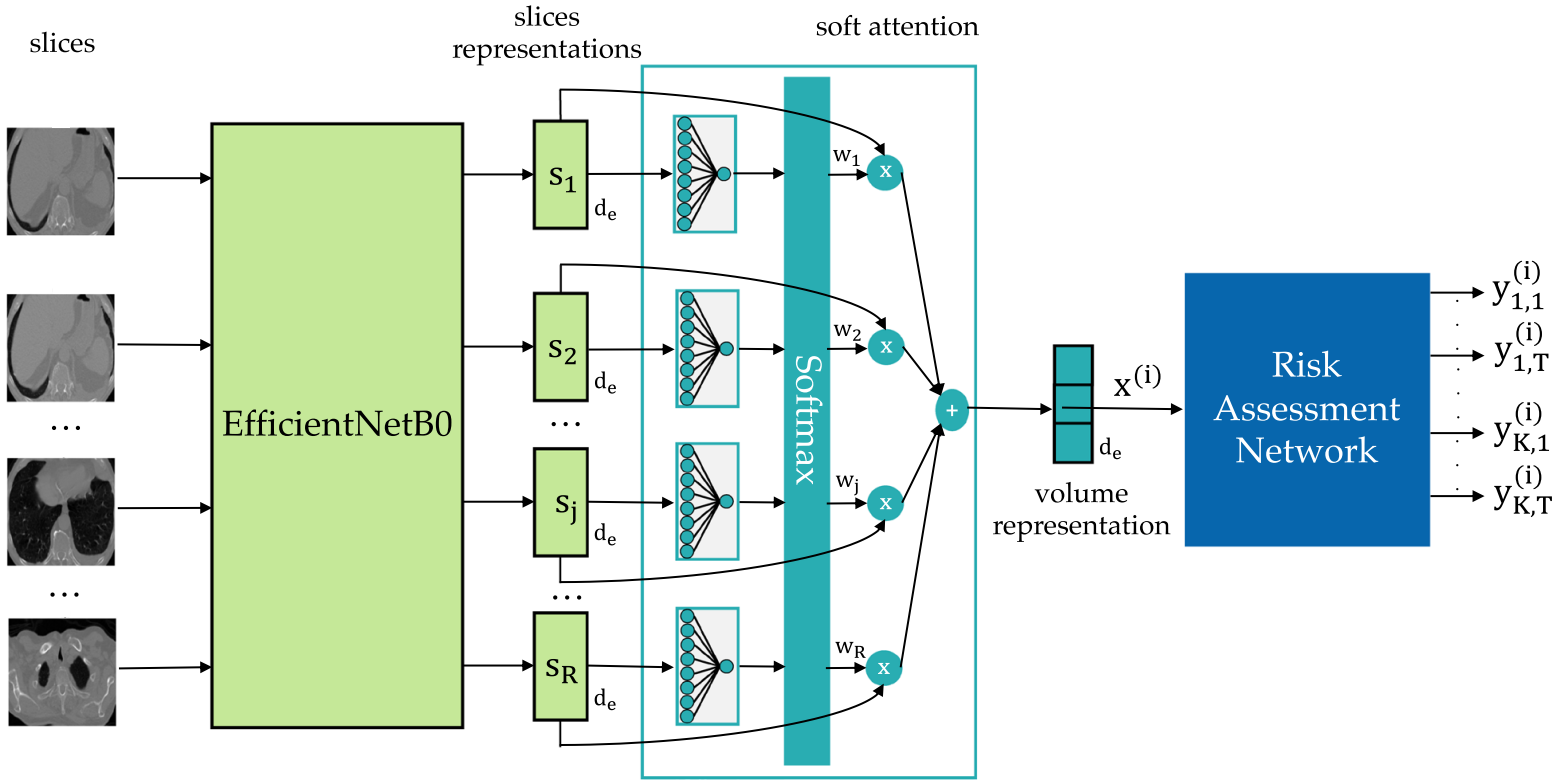
Overview of the proposed method. The approach employs the EfficientNetB0 as the backbone to generate dense representations of CT slices within a volume. These representations are then combined using a soft attention mechanism to produce a comprehensive 3D representation. This 3D representation is subsequently processed by the risk-assessment network to predict survival risk

## Methods

We propose a novel architecture for 2-year OS risk prediction integrating a 2D CNN, specifically EfficientNetB0, with a soft attention mechanism. This combination creates a comprehensive representation of the CT volume, which is then processed through a risk-assessment network called DeepHit. Our method is depicted in Fig. 2 and presented in the following subsections.

### Problem formulation

Survival data for each patient provides three critical elements: the observed features, the time elapsed since features were collected, and a label indicating whether the event of interest (e.g., death) has occurred. In our approach, we consider survival time as a discrete number, with a finite time horizon. We define the time set as $T = \{0, \ldots , T_{\text {max}}\}$, where $T_{\text {max}}$ represents the predetermined maximum time horizon, set to 2 years with a monthly granularity, resulting in $T_{max} = 24$.

A common challenge in survival data is truncation, which occurs when the event of interest is not observed due to the patient being lost to follow-up. Specifically, truncation happens when the observation period for a patient ends before the event (e.g., death) occurs. Addressing this challenge is a crucial aspect of our analysis. We define *truncation* as the event $k=0$ and the set of possible events as $K = \{0, 1\}$, where $k=1$ denotes the event of interest. Each data point is thus therefore a triple $(\textbf{x}, s, k)$, where $\textbf{x} \in \mathbb {R}^D$ is a *D*-dimensional vector of features, $s \in T$ is the time at which the event or truncation occurred, and $k \in K$ indicates whether the event ($k=1$ ) or truncation ($k=0$) occurred at time *s*.

The dataset $D = \{(\textbf{x}^{(i)}, s^{(i)}, k^{(i)})\}_{i=1}^N$ describes a finite set of observed instances or patients in our analysis. For each tuple $(\textbf{x}^{(i)}, s^{(i)}, k^{(i)})$ with $k^{(i)} \ne 0$, our focus is on determining the actual probability $P(s = s^{(i)}, k = k^{(i)} \mid \textbf{x} = \textbf{x}^{(i)})$, which models the likelihood that a patient with features $\textbf{x}^{(i)}$ will encounter the event $k^{(i)}$ at time $s^{(i)}$. Since the true probabilities cannot be directly derived from a finite dataset, our objective is to estimate $\hat{P}$ as an approximation of these probabilities, providing a reliable representation of the underlying survival dynamics.

### EfficientNetB0 and soft attention mechanism

Our approach harnesses the power of automatic feature extraction from images while focusing on the most relevant slices within the 3D CT scans, thanks to the soft attention mechanism. We selected the EfficientNet-B0 as our backbone due to its numerous advantages. The primary benefit is its exceptional efficiency. EfficientNet-B0 consists of a low number of parameters and FLOPS (Floating Point Operations per Second), detailed in Section "Experimental setup" This ensures that we can obtain high-quality features while minimizing the number of parameters, reducing the risk of overfitting on small datasets. Moreover, EfficientNet-B0 is a versatile network. Its efficient feature extraction capabilities make it suitable for a wide range of computer vision tasks, enhancing its applicability across different projects [27–30]. The EfficientNetB0 was pretrained on ImageNet [31] and only the last layer was fine-tuned for the OS risk prediction task. For each slice *j* of a volume *i* the EfficientNetB0 generates a representation $\mathbf {s_{j}}^{(i)}$ of size $d_e$. These representations are then combined using the soft attention mechanism by assigning each of them a weight, which is a scalar obtained by applying the softmax function to the output of a fully connected layer (soft attention mechanism):

$$\begin{aligned} w_j^{(i)} = \frac{\text {exp}\left( {\textbf {W}} {\textbf {s}}_j^{(i)} + b\right) }{\sum _{k=1}^R{\text {exp}\left( {\textbf {W}} {\textbf {s}}_k^{(i)} + b\right) }}, \quad i = {1, \dots , R} \end{aligned}$$

where ${\textbf {W}} \in \mathbb {R}^{1 \times d_e}$ and ${\textbf {b}} \in \mathbb {R}$ are learnable parameters. The result is a representation $\textbf{x}^{(i)}$ of size $d_e$ that summarizes the information from the volume, focusing on the most important slices.

1 $$\begin{aligned} \textbf{x}^{(i)} = \sum _{j=0}^{R} w^{(i)}_{j}\textbf{s}^{(i)}_{j} \end{aligned}$$

where *R* is the number of slices in the volume *i*, $w^{(i)}_{j}$ is a scalar weighing the contribution of *j*-th slice and $\textbf{s}^{(i)}_{j}$ is the *j*-th slice representation of *i*-th volume. Hence, this method provides a 3D representation of the CT scan using a 2D CNN with few parameters (328.224) and a soft attention mechanism, thereby reducing the model’s complexity compared to a 3D CNN. This reduction in complexity leads to easier training and better generalization, especially when data is limited, as in our case.

### Risk-assessment network

The representation $\textbf{x}^{(i)}$ becomes the vector of features fed to a risk-assessment neural network named DeepHit [22]. It is important to note that no clinical or interventional data were used in the analysis; all DeepHit predictions were exclusively based on imaging features extracted from the baseline pre-treatment CT scans. DeepHit learns the distribution of survival times directly, without making assumptions about the form of the underlying stochastic process. The objective is to train the risk-assessment network to acquire the knowledge of $\hat{P}$, the estimate for the joint distribution of survival times and competing events. DeepHit consists of a shared sub-network (SN) and multiple cause-specific sub-networks (CSNs), depending on the number of events *k*. To ensure learning of the joint distribution of *k* competing events, rather than the marginal distributions of individual events, DeepHit employs a single softmax layer as its output layer. Additionally, the model incorporates a residual connection linking the input features to each CSN, enhancing the robustness and effectiveness of the learning process. In our specific context, the sole event under consideration is the patient’s death, denoted as $k=1$. Consequently, we have just one CSN. The shared SN and the CSN are composed of fully-connected layers. The shared SN takes features $\textbf{x}^{(i)}$ as inputs and generates an output vector $f_s(\textbf{x}^{(i)})$ capturing the latent representation of features. On the other hand, the CSN takes pairs $z = (f_s(\textbf{x}^{(i)}), \textbf{x}^{(i)})$ as inputs and produces an output vector $f_c(z)$ representing the probability of the survival times. These SNs incorporate both the output of the shared network and the original features as inputs. This design allows the SNs to access the learned common representation $f_s(\textbf{x}^{(i)})$ while retaining the ability to learn distinct aspects of the representation. The softmax layer generates a probability distribution denoted as $\textbf{y}=[y_{1,1},...,y_{1,T_{max}}]$, where $y_{1,s}$ represents the estimated probability $\hat{P}(s,k^{(i)})|\textbf{x}^{(i)})$ indicating the likelihood of the patient experiencing event $k^{(i)}=1$ at the time *s*. This architectural framework enables the network to capture potentially non-linear and non-proportional relationships between features and associated risks. To assess the risk of event occurrence, the cause-specific cumulative incidence function (CIF), expressed as $F_{k^{(i)}}(t|\textbf{x}^{(i)}$), is employed. This function quantifies the probability that the event $k^{(i)}=1$ occurs by time *t*, given the features $\textbf{x}^{(i)}$, where *t* represents the time horizon, set to 2 years in our case. Formally, the CIF for event $k^{(i)}=1$ is expressed as:

2 $$\begin{aligned} F_{k^{(i)}}(t|\textbf{x}^{(i)}) = \sum _{s^{*}=0}^{t} P(s=s^{*},k=k^{(i)}|\textbf{x}=\textbf{x}^{(i)}). \end{aligned}$$

However, since the true CIF $F_{k^{(i)}}(t|\mathbf {x^{(i)}})$ is not known, we utilize the estimated CIF:

3 $$\begin{aligned} \hat{F}_{k^{(i)}}(s^{(i)}|\textbf{x}^{(i)}) = \sum _{m=0}^{s^{(i)}} y_{1,m}. \end{aligned}$$

### Loss function

We used the loss function $\mathcal {L}$ of DeepHit, specifically crafted to handle truncated data effectively. It is expressed as $\mathcal {L} = \alpha \mathcal {L}_1 + \beta \mathcal {L}_2$, where $\alpha$ and $\beta$ are weights for the terms $\mathcal {L}_1$ and $\mathcal {L}_2$, described as follows. The term $\mathcal {L}_1$ represents the log-likelihood of the joint distribution of the first hitting time and the unique event, adapted to accommodate truncated data. For patients who have not experienced truncation, $\mathcal {L}_1$ includes both the occurrence of the event and the corresponding time. For patients who have been truncated, $\mathcal {L}_1$ captures the truncation time, indicating they were alive up to that point, thus providing valuable status information. Formally:

4 $$\begin{aligned} \begin{aligned} \mathcal {L}_1 = -\sum _{i=1}^{N} \left[\mathbbm{1}(k^{(i)} \ne \emptyset ) \cdot log(y_{k^{(i)},s^{(i)}}^{(i)})\right] \\ + \mathbbm{1}(k^{(i)} = \emptyset ) \cdot log\left(1 - \sum _{k=1}^{K} \hat{F}_{k}(s^{(i)}|\textbf{x}^{(i)})\right) , \end{aligned} \end{aligned}$$

where $\mathbbm{1}$ is an indicator function and *N* is the number of patients in the dataset. The first term captures information from patients who have not undergone truncation, whilst the second term addresses truncation bias by recognizing that these patients are alive at truncation time, enabling the model to anticipate that the first hitting event will occur after this time.

The $\mathcal {L}_2$ term incorporates cause-specific ranking loss functions, utilizing the estimated CIFs computed at various times corresponding to the instances when events occur. This approach fine-tunes the network for each cause-specific estimated CIF. The ranking loss function integrates the concept of concordance: a patient experiencing an event at time *s* should exhibit a higher risk at time *s* than a patient who has survived beyond *s*. Formally:

5 $$\begin{aligned} \mathcal {L}_2 = \sum _{k=1}^{K} \theta _k \cdot \sum _{\begin{array}{c} i=1 \\ i \ne j \end{array}}^{N} A_{k,i,j} \cdot \eta (\hat{F}_k(s^{(i)}|\textbf{x}^{(i)}), \hat{F}_k(s^{(i)}|\textbf{x}^{(j)})), \end{aligned}$$

where the coefficients $\theta _k$ are chosen to balance the ranking losses of the *k*-th competing event, $\eta (a,b)$ is a convex loss function defined as $\eta (a, b) = \exp \left( -\frac{(a - b)}{\sigma } \right)$ with $\sigma$ set to 0.1, and $A_{k,i,j}$ is defined as:

6 $$\begin{aligned} A_{k,i,j} = \mathbbm{1}(k^{(i)}=k, s^{(i)}<s^{(j)}), \end{aligned}$$

representing pairs (*i*, *j*) suitable for event *k*. The inclusion of $\mathcal {L}_2$ in the overall loss function penalizes the misordering of pairs concerning each event. Consequently, minimizing the total loss encourages the correct ordering of pairs for each event.

## Experimental setup

This section elucidates the experiments conducted to evaluate the effectiveness and quality of our approach. Additionally, it elaborates on the experimental setting and the performance metric employed for these evaluations.

### Comparative analysis

To demonstrate the benefits and strengths of our methodology, we compared our model against several competitors, each selected and configured to provide a clear and rigorous comparison. Below, we outline the key questions we aim to address through these comparisons, along with a detailed description of the experimental setups for each competing method.

*Question 1: How does our method compare to 3D networks?*

To assess the effectiveness of our approach for constructing a robust representation of 3D volumes for the 2-year OS risk prediction task, we carried out a comparative evaluation by replacing our approach with competitive 3D baselines: an 18-layer 3D ResNet (ResNet3D_18) [32] pre-trained on the Kinetics dataset [33], a 121-layer DenseNet3D (DenseNet3D_121), and a Medical Slice Transformer (MST) [34] leveraging DINOv2 [35]. We evaluated both the 3D networks and our approach by employing different layer-freezing strategies. We evaluated two training strategies. First, we froze half of the layers in both the 3D networks and the 2D backbone (EfficientNetB0) of our approach. Next, we opted to freeze all layers except the last one (feature extractor), based on the hypothesis that reducing the number of parameters to optimize would enhance generalization with a limited dataset. This systematic evaluation allowed us to understand the impact of various layer-freezing configurations on the performance and efficiency of the models.

*Question 2: How effective is the soft attention mechanism?*

We aimed to demonstrate the efficacy of the attention mechanism by comparing it with two alternative approaches: a simple average of the slices representations generated by the backbone, and a self-attention mechanism that incorporates a class token (CTk) summarizing the entire volume before being passed to the risk-assessment network. This set of experiments was conducted by freezing all backbone layers except the last one, as this configuration yielded higher performance in previous experiments.

*Question 3: Is our approach adaptable to different 2D backbones?*

To illustrate the adaptability of our approach with various 2D backbones, we conducted another set of experiments using as backbone widely recognized architectures, including VGG16 [36], ResNet50 [37], AlexNet [37], Vision Transformer (ViT) [38], and MedViT_small [39]. These architectures were selected to represent different foundational paradigms—namely, convolutional networks (e.g., VGG16, ResNet50, AlexNet) and attention-based models (e.g., Vision Transformer, MedViT)—to evaluate the versatility of our approach across both convolutional and attentional frameworks. This set of experiments also was conducted by freezing all backbone layers except the last one.

*Question 4: What is the impact of fine-tuning?*

To underscore the significance of transfer learning, particularly when dealing with limited data, we conducted two experiments using the CLARO dataset. One experiment involved training without fine-tuning on LUNG1, whilst the other included fine-tuning on LUNG1.

*Question 5: What is the computational complexity of the proposed method?*

To demonstrate the efficiency and cost-effectiveness of our approach, we conducted a computational complexity analysis of the various neural network configurations by considering the mean number of slices in the dataset, i.e. 72 and includes the number of parameters and the FLOPS for each configuration, providing insight into the computational demands of each model.

**Table 2:** Hyperparameters of the risk-assessment network

Hyperparameter	Value
# hidden layers for both SN and CSNs	5
# neurons per hidden layer	100
Dropout rate	0.2
Activation function	ReLU
Loss function $\mathcal {L}$	
$\alpha$	0.5
$\beta$	0.5

### Performance metric

We used the time-dependent concordance index ($C^{td}$-index) [40] as our performance metric, which ranges from 0 to 1. It is important to highlight that the conventional concordance index (*C*-index) [41] is a widely utilized discriminative metric. The *C*-index operates under the assumption that patients with longer lifespans should be associated with a lower risk compared to those with shorter lifespans. However, the traditional *C*-index is calculated solely at the initial observation time, lacking the capacity to capture potential variations in risk over time. In contrast, the time-dependent concordance index considers the temporal aspect, offering a more comprehensive understanding of how risk evolves over the course of observation. The $C^{td}$-index for event *k* is defined as:

7 $$\begin{aligned} \begin{aligned} C^{td} = P(\hat{F}_k(s^{(i)}|\textbf{x}^{(i)})> \hat{F}_k(s^{(i)}|\textbf{x}^{(j)})|s^{(i)}<s^{(j)}) \\ \approx \frac{\sum _{i \ne j} A_{k,i,j} \cdot \mathbbm{1}(\hat{F}_k(s^{(i)}|\textbf{x}^{(i)})>\hat{F}_k(s^{(i)}|\textbf{x}^{(j)}))}{\sum _{i \ne j} A_{k,i,j}}. \end{aligned} \end{aligned}$$

Thus, the $C^{td}$-index for event *k* is computed by comparing pairs of observations. In each pair, one patient has experienced event *k* at a specific time, whilst the other has neither encountered the event nor been censored by that time. The significance of this discriminative index lies in its independence from a single fixed time. This characteristic renders it well-suited for situations where the impact of covariates on survival varies over time. In other words, this index is particularly valuable when risks exhibit non-proportional behavior throughout the observation period. Hence, the $C^{td}$-index evaluates how well a model ranks patients according to their risk over time. When a single type of input data is used, models often capture similar patterns, which can result in $C^{td}$ values that are close to each other. Nevertheless, even small differences in the index can indicate meaningful distinctions in predicted risk, helping to identify patients who may require closer monitoring or different management strategies.

### Experimental settings

We implemented all considered methods in PyTorch [42] and trained them on an NVIDIA A100 40GB GPU. The training data augmentation strategy involved randomly flipping and rotating within a range of 20$^\circ$ each 2D slice with a probability of 0.2. During the training and evaluation of the risk-assessment network, we employed tenfold cross-validation. For each fold, the 90% portion allocated for training was further split using a 90-10 ratio (90% for training and 10% for validation). All tested networks were trained with AdamW optimizer with base learning rate $1 \times 10^{-4}$, weight decay $1 \times 10^{-2}$ and batch size 4 for 100 epochs. The best-performing model configuration was selected based on the highest time-dependent concordance index ($C^{td}$-index) achieved on the validation set. The loss weights and the other DeepHit hyperparameters were not optimized but were kept fixed at the values listed in Table 2. For the experiments on the CLARO dataset, we selected the best model configuration for each fold based on performance on the LUNG1 validation set. We then fine-tuned this configuration using the CLARO training set.

## Results and discussion

We conducted a series of experiments to thoroughly evaluate the performance and flexibility of our proposed approach. First, we aimed to compare it against 3D baselines in survival prediction tasks, particularly focusing on the impact of employing a soft attention mechanism. Additionally, we tested the adaptability of our method by integrating alternative 2D backbones, such as ResNet50 and ViT, to assess whether it can maintain strong performance across diverse architectures. Moreover, we explored the significance of domain-specific transfer learning by fine-tuning models pre-trained on the LUNG1 dataset to investigate its impact on the smaller CLARO dataset and performed a detailed computational complexity analysis to determine the efficiency of our approach and its viability for survival prediction tasks in resource-limited environments. In the following, we present the results addressing the questions raised in Section "Comparative Analysis".

**Table 3:** Comparison between our approach and ResNet3D_18

Approach	Training strategy	$C^{td}$-index (mean ± std)
ResNet3D_18	Half layers freezed	$0.528 \pm 0.069$
DenseNet3D_121	Half layers freezed	$0.5331 \pm 0.038$
MST	Half layers freezed	$0.4915 \pm 0.066$
EfficientNetB0 + Soft Attention	Half layers freezed	$0.572 \pm 0.064$
ResNet3D_18	Feature extractor	$0.507 \pm 0.074$
DenseNet3D_121	Feature extractor	$0.5281 \pm 0.056$
MST	Feature extractor	$0.504 \pm 0.051$
**EfficientNetB0 + Soft Attention**	Feature extractor	${\textbf {0.584}} \pm {\textbf {0.054}}$

Results of $C^{td}$-index over tenfold cross-validation

The best result is highlighted in bold

*Question 1: How does our method compare to 3D networks?*

Table 3 summarizes the results of the initial set of experiments, where our approach was compared with competitive 3D baselines: an 18-layer ResNet3D, a 121-layer DenseNet3D, and a Medical Slice Transformer.

As shown in the table, our fusion approach, which combines EfficientNetB0 with a soft attention mechanism, consistently outperforms the 3D models under both layer-freezing strategies. This demonstrates the superior capacity of our method to create a consistent volume representation suitable for the OS prediction task, which is crucial for the risk-assessment network. Notably, the best performance is achieved with the EfficientNetB0 as the feature extractor, indicating that optimizing fewer parameters leads to better generalization when dealing with limited data availability. Given that we only one measurement per fold in the cross-validation, classical significance tests such as the paired t-test or Wilcoxon signed-rank test are not reliable, as their statistical power is limited in such low-sample settings and their distributional assumptions may not hold. To address this, we adopted a permutation-based paired t-test. In this approach, the observed difference in $C^{td}$-index between two models is compared against a null distribution obtained by repeatedly (5000 times) randomly swapping (with probability 0.5) the predictions of the two models at the patient level. The *p*-value is then computed as the proportion of permuted differences as extreme as the observed one. This non-parametric procedure makes no assumptions about the underlying distribution and is therefore more robust in our context of limited sample size. The results show that our proposed method (EfficientNetB0 + Soft Attention) achieves statistically significant improvements compared to all 3D baselines (ResNet3D_18, DenseNet3D_121, and MST), with $p < 0.05$ in all cases. This confirms that the observed performance gains are unlikely to be due to chance. The only non-significant comparison ($p = 0.4162$) arises when comparing our approach trained with the *feature extractor* strategy against the same model trained with *half of the layers frozen*. This result highlights that the advantage comes not only from the architecture itself but also from the training strategy: using EfficientNetB0 as a frozen feature extractor reduces the number of trainable parameters and thus improves generalization under limited data availability.

**Table 4:** Comparison between the soft attention mechanism with the Average Pooling and Self Attention approaches

Approach	Aggregation mechanism	$C^{td}$-index (mean ± std)
EfficientNetB0	Average pooling	$0.570 \pm 0.033$
EfficientNetB0	Self attention + CTk	$0.579 \pm 0.079$
**EfficientNetB0**	Soft attention	${\textbf {0.584}} \pm {\textbf {0.054}}$

All backbone layers are frozen, except for the last one (feature extractor). Results of $C^{td}$-index over tenfold cross-validation

The best result is highlighted in bold

*Question 2: How effective is the soft attention mechanism?*

We compared the soft attention approach with a simple average of the slices’ representations generated by the backbone, and a self-attention mechanism incorporating a class token (CTk) summarizing the entire volume. We selected in all the experiments the EfficientNetB0 as feature extractor since it was the experiment with the best performance in the previous analysis in Section "Results and Discussion". Table 4 shows the results of this comparison study.

The table demonstrates that the fusion approach using the soft attention mechanism yields superior performance compared to both the average and self-attention approaches. The superior performance of the soft attention mechanism over the Average Pooling approach can be attributed to its ability to weigh the most important slices within the volume. This weighting allows the risk-assessment network to focus on the most relevant portions of the volume, which are more critical for overall survival prediction. When compared to the self-attention mechanism, the superior performance of soft attention may be due to its better generalization capability and the optimization of fewer parameters, which together can lead to more effective learning and improved performance, especially when dealing with limited data, as shown in the previous experiments. To assess the statistical significance of these differences, we employed a permutation-based paired t-test across the 10 cross-validation folds. The results confirmed that the improvements of soft attention over average pooling ($p = 0.5450$) and over self-attention ($p = 0.3240$) were not statistically significant. Nevertheless, the proposed method consistently achieved the highest mean $C^{td}$-index across folds, supporting its robustness. Beyond the numerical results, soft attention provides a more flexible aggregation strategy by weighting slices according to their relevance, providing interpretable insights into which regions contribute most to the prediction. In addition, compared to the self-attention mechanism, soft attention requires fewer parameters and is computationally more efficient, making it more suitable in scenarios with limited data and for practical deployment.

**Table 5:** Comparison between the EfficientNetB0 with other 2D backbones in combination with the Soft Attention mechanism

Approach	$C^{td}$-index (mean ± std)
ResNet50 + Soft Attention	$0.564 \pm 0.067$
Vgg16 + Soft Attention	$0.524 \pm 0.063$
AlexNet + Soft Attention	$0.550 \pm 0.0510$
ViT_b_16 + Soft Attention	$0.546 \pm 0.043$
MedViT_small + Soft Attention	$0.5482 \pm 0.056$
**EfficientNetB0 + Soft Attention**	${\textbf {0.584}} \pm {\textbf {0.054}}$

All backbone layers are frozen, except for the last one (feature extractor). Results of $C^{td}$-index over tenfold cross-validation

The best result is highlighted in bold

*Question 3: Is our approach adaptable to different 2D backbones?*

Table 5 reports the results of these experiments. EfficientNetB0’s superior performance with respect to other 2D backbones stems from its compound scaling method, which balances network depth, width, and resolution, optimizing its architecture for both accuracy and efficiency. Additionally, its streamlined architecture, characterized by a reduced number of parameters, makes it particularly well-suited for data-limited scenarios, where it effectively minimizes the risk of overfitting while preserving high computational efficiency. As evidenced by the results, our fusion approach achieves higher performance with different backbones compared to the 3D approach. This demonstrates that the mechanism of combining 2D slice representations to construct a 3D volume representation is more effective for OS prediction, particularly when working with limited data. The enhanced performance underscores the advantage of leveraging 2D networks over directly using 3D networks in this context.

**Table 6:** Results of $\mathbf{C}^{\mathbf{td}}$-index on CLARO

Approach	Fine-tuning on LUNG1	$C^{td}$-index
EfficientNetB0 + Soft Attention	No	0.503
**EfficientNetB0 + Soft Attention**	Yes	${\textbf {0.579}}$

All backbone layers are frozen, except for the last one (feature extractor)

The best result is highlighted in bold

*Question 4: What is the impact of fine-tuning?*

The results of these experiments are presented in Table 6. Since the CLARO dataset is extremely small, we did not report the standard deviation. We had to aggregate the test folds to obtain a single measurement, making it impossible to calculate variability across folds. The results clearly indicate that domain-specific transfer learning from the LUNG1 dataset leads to better performance on the CLARO dataset compared to fine-tuning. To further demonstrate the robustness of our approach, we repeated the experiments five additional times and performed statistical testing. With fine-tuning on LUNG1, the model achieved an average performance of $0.577 \pm 0.021$, whereas training a model from scratch directly on the CLARO dataset resulted in $0.503 \pm 0.017$.

To compare the two models we conducted a paired statistical test. Each model was evaluated over the six repeated runs, with each run producing a single aggregated performance metric from a tenfold cross-validation. The pairing is feasible because the same data splits and experimental conditions were used for both models in each run, ensuring that performance differences reflect the model initialization strategy rather than variability in the data.

Given the small number of paired samples ($n=6$) and the unknown distribution of performance differences, we used the Wilcoxon signed-rank test, a non-parametric alternative to the paired t-test. This test assesses whether the median difference between paired observations is significantly different from zero without assuming normality. Additionally, we report the effect size (r) to quantify the magnitude of the observed difference.

The Wilcoxon signed-rank test yielded a statistic of 0.0, with a p-value of 0.03125 and a large effect size (r = 0.899).

These results indicate a statistically significant difference between the two models ($p < 0.05$), with the pretrained model consistently outperforming that trained from scratch. The large effect size ($r \approx 0.9$) further confirms that this difference is not only statistically significant but also practically meaningful.

In conclusion, pretraining the EfficientNetB0-based architecture provides a clear advantage over training from scratch, and the Wilcoxon signed-rank test robustly supports this finding despite the limited number of repeated runs.

This improvement can be attributed to the limited data available in the CLARO dataset, which makes fine-tuning a model from scratch challenging. In contrast, leveraging the knowledge learned from the LUNG1 dataset provides a substantial boost, as the pretrained model can exploit previously acquired patterns and features, thereby enhancing performance on the smaller CLARO dataset. These findings underscore the value of domain-specific transfer learning in scenarios with constrained data availability.

In addition, to further assess the generalizability of our approach and examine robustness to domain shift, we directly evaluated the best-performing model (pretrained on LUNG1) selected from the tenfold cross-validation on the external CLARO dataset without any fine-tuning. The model achieved a $C^{td}$-index of 0.5608, indicating that the learned representations retain prognostic relevance beyond the training cohort, despite the absence of dataset-specific adaptation.

Looking ahead, future work will further strengthen generalizability by validating the model on additional larger external cohorts beyond CLARO, thereby addressing potential limitations related to sample size and overfitting.

**Fig. 3:**
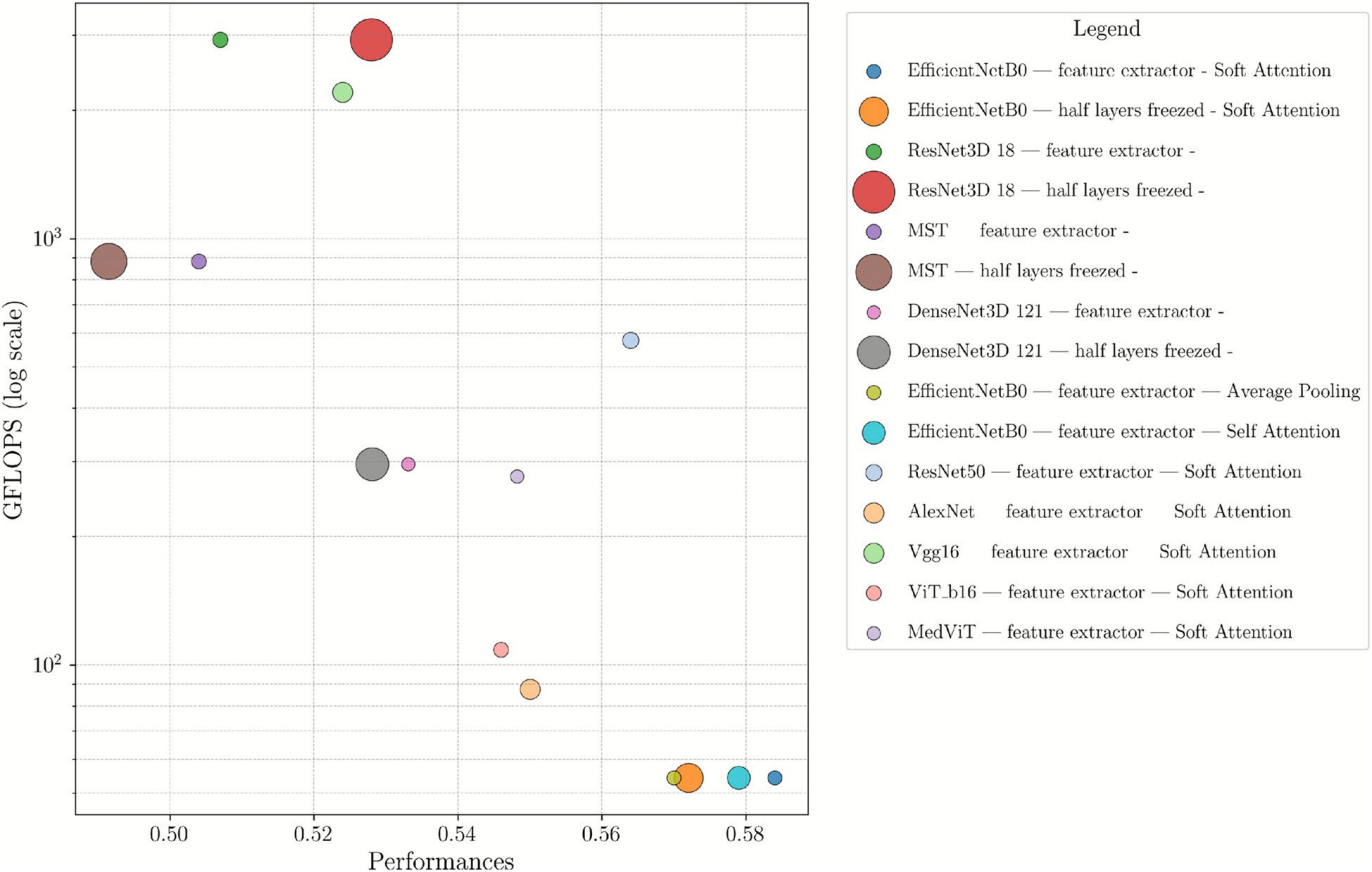
Computational complexity analysis of the experimented models. The x-axis represents the model’s performance, while the y-axis shows the number of giga floating-point operations per second (GFLOPS) required for inference. The size of each point is proportional to the number of parameters of the corresponding model, providing a visual representation of the trade-off between model accuracy, computational cost, and model size

*Question 5: What is the computational complexity of the proposed method?*

The computational complexity analysis of the various model configurations is detailed in Fig. 3. The figure is a bubble chart comparing various model configurations based on their performances (x-axis) and GFLOPS (y-axis, log scale). Each bubble represents a specific model, with its size indicating the number of parameters. The analysis highlights the trade-offs between performance and computational cost across different model architectures and layer-freezing strategies. Notably, EfficientNetB0 variants are characterized by lower parameter counts and computational costs, making them a suitable choice for resource-constrained environments with high performance. In the versions of EfficientNet with the lowest costs, the soft attention mechanism is utilized to further enhance efficiency. In comparison, other 2D backbones demonstrate inferior performance and higher computational costs relative to EfficientNetB0, underscoring the crucial role of parameter efficiency in data-limited scenarios. On the other hand, 3D networks yields significantly higher computational costs and parameter counts, leading to lower efficiency and performance.

## Interpretability

**Fig. 4:**
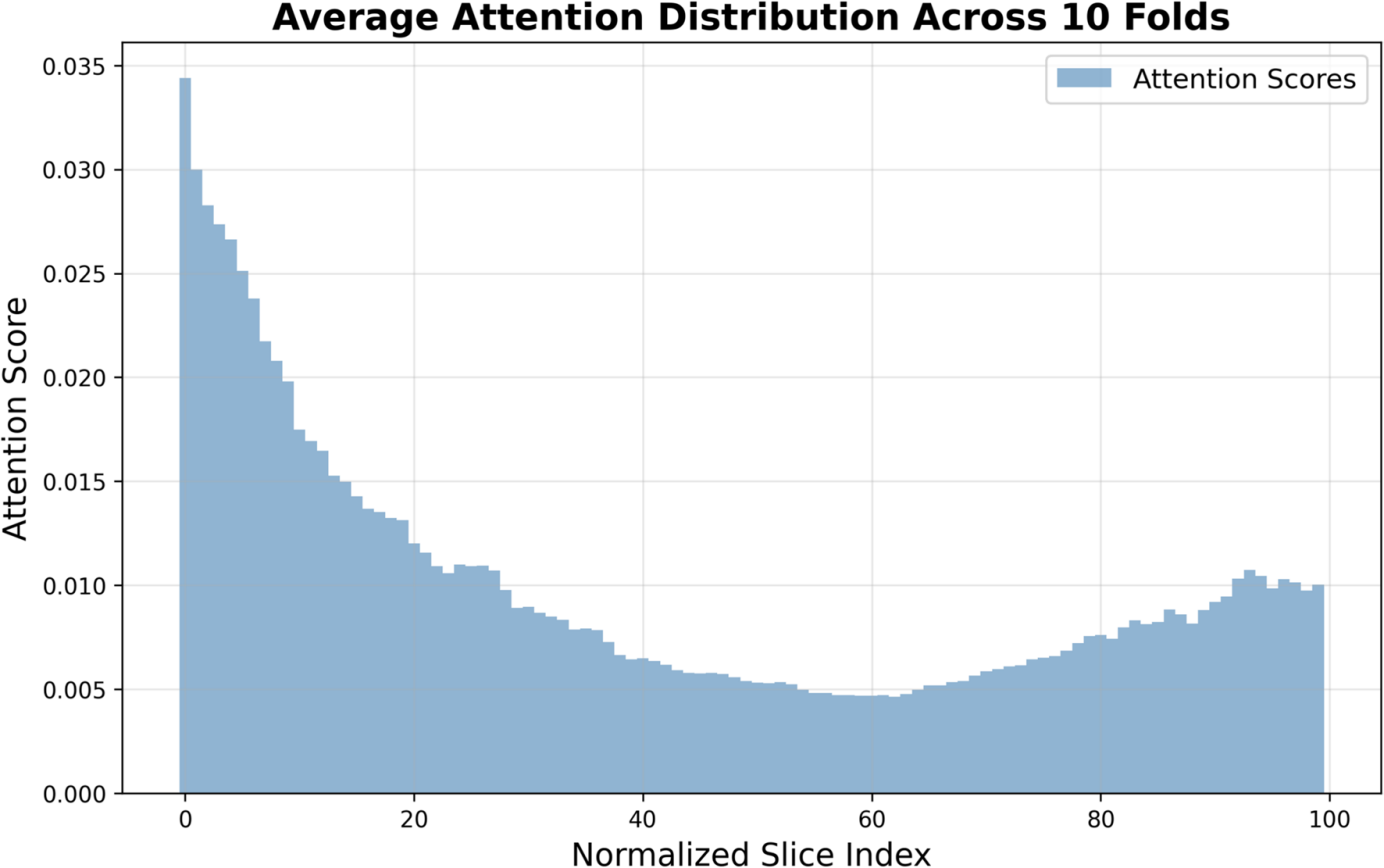
Average attention weight distributions generate by our model

Interpretability remains a fundamental challenge in deep learning, particularly in medical applications: understanding why a model produces a specific prediction is critical for clinical trust and adoption. In this study, we address this challenge by leveraging attention weights derived from a soft attention mechanism to identify the CT scan slices that the model considered most informative for prognostic prediction. By highlighting these slices, we provide a window into the model’s decision-making process, thereby bridging the gap between algorithmic output and clinical understanding.

Our model was trained to assign attention weights to individual slices within a 3D CT volume, quantifying their relative contribution to the final prognostic outcome. For the interpretability analysis, we relied on the models trained on the LUNG1 dataset in the 10 cross-validation folds. For each fold, attention weights were computed on the corresponding test set. Since the number of slices varies between patients, we normalized slice positions by calculating relative slice indices. Subsequently, we averaged the attention weights across all patients and folds to obtain a robust, mean attention distribution.

Analysis of this aggregated distribution revealed a consistent and clinically meaningful pattern, as illustrated in Fig. 4. Specifically, basal lung regions consistently received higher attention weights from the model. This observation aligns closely with clinical evidence: tumors located in the basal regions of the lung are often associated with worse outcomes in non-small cell lung cancer. Basal tumor location has been linked to several prognostic factors, including:

- Advanced stage and aggressiveness: Tumors in basal regions are frequently detected at later stages and may exhibit higher invasive potential due to delayed symptom onset and the complexity of anatomical positioning [43–45].
- Proximity to vital structures: Basal tumors are anatomically positioned near critical organs such as the heart, great vessels, and diaphragm, which can complicate surgical interventions and directly impact prognosis.
- Increased symptom burden: These tumors often result in more severe respiratory impairment and cardiac complications, including pleural effusion and atelectasis, negatively affecting both survival and quality of life.

The correspondence between the model’s attention focus and established clinical knowledge reinforces the biological plausibility of the learned features. This not only enhances interpretability but also increases confidence in the model’s ability to capture clinically relevant patterns, which is essential for translational applications in precision oncology. By highlighting CT slices associated with known prognostic indicators, attention-based interpretability enables a more transparent and informative dialogue between AI predictions and clinician decision-making.

**Fig. 5:**
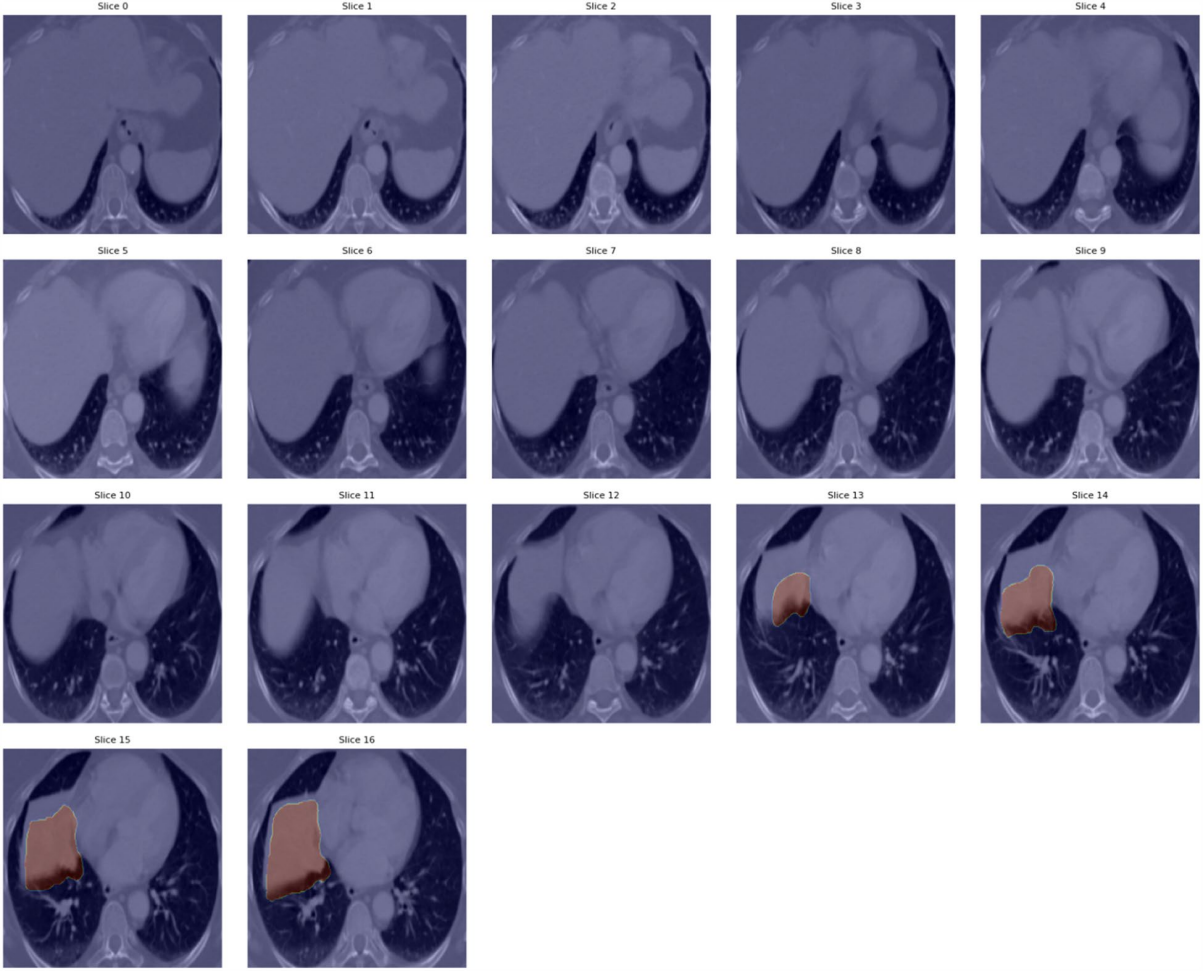
Example of the attention weight distribution for patient LUNG1-040 (stage III NSCLC, total slices = 57). Slices around the 50th percentile of the mean attention distribution are highlighted, revealing a concentration of attention in the basal lung regions corresponding to the tumor site. This pattern aligns with the advanced disease stage. In this case, the lesion (NSCLC, large cell type) is challenging to visually identify on CT images due to its low contrast, indistinct margins, and proximity to the diaphragm, underscoring the value of automated models in detecting subtle imaging patterns

An illustrative example is shown in Fig. 5, highlighting the slices in the 50th percentile of the mean attention weight distribution for patient LUNG1-040 (total slices = 57) with stage III NSCLC. The tumor is present in the lower (basal) slices, consistent with its advanced stage and basal position, which underscores the model’s ability to capture clinically meaningful spatial patterns in the lung. Furthermore, the patient’s observed survival time was 19 months, and the model assigns a corresponding probability of 0.61 for the occurrence of the event “death” at that time, demonstrating coherent alignment between model prediction and clinical outcome.

## Conclusions

In this study, we propose an innovative method for OS prediction in NSCLC using a pre-trained 2D CNN EfficientNetB0 to generate rich representations of 3D volumes. Our approach integrates a soft attention mechanism to enhance the accuracy of survival predictions, outperforming other approaches such as 3D network, average pooling, and self-attention mechanism. These results demonstrate the effectiveness of our method in predicting OS in NSCLC. While the observed $C^{td}$-index of 0.584 is only modestly above random chance (0.5), it demonstrates that meaningful prognostic information can be extracted from imaging alone. This underscores the potential of our approach as a methodological framework that may be extended in future research to other clinical prediction tasks, contingent on broader validation. We also demonstrate that a transfer learning strategy involving pre-trained models can improve performance on limited datasets. As a first step toward broader external validation, we also evaluated on CLARO the pretrained model directly on LUNG1 without fine-tuning, obtaining a $C^{td}$-index of 0.5608. This result supports the robustness of our approach, even in the absence of domain-specific adaptation, and motivates future extensions to additional large-scale external cohorts. To further ensure generalizability, future work will extend validation to additional larger external cohorts beyond CLARO, addressing concerns related to sample size and overfitting, explicitly investigating the impact of scanner type, acquisition parameters, and center heterogeneity to better understand and mitigate potential domain shift. Beyond the achievements highlighted in this study, our methodology can be tailored to tackle a broader spectrum of clinical prediction tasks. It has the potential to be applied to various medical domains, such as predicting disease progression, treatment response, or patient prognosis across different conditions. While the current study focused exclusively on imaging data to rigorously evaluate the contribution of our architectural innovations, the framework is fully compatible with hybrid and multimodal models. In future work, we aim to extend the methodology to integrate clinical variables, treatment information, and diverse data modalities beyond CT scans, including both structured and unstructured sections of electronic health records, which are expected to further improve survival prediction and patient stratification in NSCLC.

In conclusion, the presented methodology not only advances the understanding of NSCLC prognosis but also lays the foundation for a wide range of clinical prediction applications. Its adaptability and potential to synergize with various data sources make it a promising tool for the future of medical research and healthcare.

## Data Availability

Link for LUNG1: https://www.cancerimagingarchive.net/collection/nsclc-radiomics
